# Leitfaden für das Management von Folgen viraler Erkrankung mit SARS-CoV-2 aus Sicht der Ergotherapie: Ergänzung zur Leitlinie S1 für das Management postviraler Zustände am Beispiel Post-COVID-19

**DOI:** 10.1007/s00508-023-02243-y

**Published:** 2023-07-31

**Authors:** Ursula M. Costa

**Affiliations:** Ergotherapie und Handlungswissenschaft, fhg – Zentrum für Gesundheitsberufe Tirol GmbH / fh gesundheit, Innrain 98, 6020 Innsbruck, Österreich

**Keywords:** Intervention, Long COVID, Alltag, Partizipation, Leitlinie, Intervention, Long COVID, Daily life, Participation, Recommendations

## Abstract

Folgen viraler Erkrankung mit SARS-CoV-2 wirken sich auf die biopsychosoziale Gesundheit und damit auf den Alltag Betroffener, deren Handlungs- und Partizipationsmöglichkeiten in sämtlichen Lebensbereichen aus. Ergotherapeut*innen verfügen in sämtlichen Versorgungsphasen über zahlreiche Möglichkeiten in der Auswahl und Gestaltung des Settings, der Interventionsmittel, -maßnahmen und -methoden, um Betroffenen im Hinblick auf größtmögliche Lebensqualität und in einer aktiven Neu-Gestaltung ihres Lebens zu unterstützen. Dieses Dokument bietet diesbezüglich einen Einblick und enthält Empfehlungen für die ergotherapeutische Praxis im Rahmen der bis zum Zeitpunkt der Verfassung verfügbaren Evidenzquellen.

## Abstract (en)

Sequelae of viral disease with SARS-CoV-2 impact clients’ biopsychosocial health, thus their daily life, with limitations resp. barriers in their occupational capacities and opportunities. Occupational therapists have numerous and, with constantly growing evidence, personalised therapeutic means, measures, and methods in any intervention phase relevant to these according health problems, supporting i. e. coping, occupational adaptation, quality of life and participatory re-shaping of (daily) life. Based on ongoing research findings and practice evidence, this document contains recommendations for occupational therapy intervention for clients with biopsychosocial health conditions post COVID-19.

## Präambel

In Bezugnahme auf die S1-Leitlinie für Primärversorger*innen bzw. Hausärzt*innen [1] ist diese Ergänzung einerseits für Berufsangehörige der Ergotherapie, andererseits für Patient*innen und deren Angehörige, sowie alle weiteren Kooperationspartner*innen – Angehörige sämtlicher Professionen und Disziplinen, die mit Betroffenen von Folgen viraler Erkrankung mit SARS-CoV‑2 arbeiten, und weitere inhaltlich Interessierte.

In Übereinstimmung mit Ersterer werden auch hier im Hinblick auf „Folgen viraler Erkrankung mit SARS-CoV-2“ Post-COVID bzw. Long-COVID adressiert [1, 2].

Grundsätzlich arbeiten Ergotherapeut*innen von der Akut- bis zur rehabilitativen Versorgung im interprofessionellen Team zur Stärkung der Handlungsfähigkeit und Eröffnung von Handlungsmöglichkeiten; sie adressieren alltagsrelevante Fähigkeiten auf Körperfunktionsebene in Verbindung mit Körperstrukturen und Aktivitäten, sowie personbezogene und umweltbezogene Kontextfaktoren im Sinne der Lebensqualität, biopsychosozialen Gesundheit und Partizipation. Dies gilt auch für Menschen nach einer COVID-19-Infektion.

Die Informationen und Empfehlungen dieser Ergänzungen zur S1-Leitlinie [1] basieren auf einer systematischen Literaturrecherche sowie auf Erfahrungswissen und Praxisevidenz des ergotherapeutischen Behandlungs- und Forschungsteams des Studiengangs Ergotherapie an der fh gesundheit unter der wissenschaftlichen und Projektleitung von Prof.^in^ Dr.^in^ Ursula Costa, MA im Rahmen des interprofessionellen und interdisziplinären PRECISE-Forschungsprojekts in Tirol unter der Leitung von Univ.-Prof.^in^ Dr.^in^ Judith Löffler-Ragg (NCT05545332); die Ausführungen sind ergänzt durch Praxiserfahrungen insbesondere aus dem Arbeitskreis von Ergotherapie Austria „Ergotherapie postCovid“ unter der Leitung von Elisabeth Semotan-Rigler (2022/23). Zusätzlich zu den folgenden Empfehlungen werden weitere versorgungsrelevante Quellen und bereits entstandenes Ergotherapie- und Gesundheitssituations-relevantes Informationsmaterial zur bestmöglichen ergotherapeutischen Versorgung im interprofessionellen Kontext empfohlen (z. B. [3–6]).

Erkenntnis- und Forschungsstand entwickeln sich laufend weiter; insofern ist dieses Dokument nicht abschließend gedacht: ein Transfer der folgenden Empfehlungen in die ergotherapeutische Praxis sollte unter Hinzuziehen und Beachtung sämtlicher jeweils aktuell relevanter Evidenzsäulen person-, kontext- und situationsbezogen erfolgen.

## Grundlegendes für die ergotherapeutische Arbeit mit Betroffenen von Folgen viraler Erkrankung mit SARS-CoV-2

Die jeweilige Gesundheitssituation und das aktuelle Setting (stationär, ambulant) sowie, nicht zuletzt, die *Handlungsbedürfnisse und Handlungsmöglichkeiten der Betroffenen* selbst entscheiden über die ergotherapeutische Indikation ebenso wie die entsprechenden ergotherapeutischen Mittel, Maßnahmen, Methoden und Konzepte.

Im Falle von Betroffenen mit Folgen viraler Erkrankung mit SARS-CoV‑2 ist der Behandlungserfolg von der *Individualisierung und situativen Anpassung des (ergo)therapeutischen Angebots* abhängig. Diese basieren auf einer *personalisierten und kontextsensiblen Herangehensweise* und bedeuten u. a. das Wahrnehmen und Erkennen von insbesondere handlungs- und alltagsrelevanten Fähigkeiten und Einschränkungen der jeweiligen Person in der aktuellen Situation.

Im Bereich der Ergotherapie für Betroffene von Folgen viraler Erkrankung mit SARS-CoV‑2 sind angesichts der vielfältigen und individuellen Auswirkungen der Viruserkrankung auf Körperfunktionen, Aktivitäten und Partizipation analog zu anderen Gesundheitsberufsangehörigen aktuelles Wissen aus und ergotherapeutische Fertigkeiten in folgenden Fachbereichen angefragt: Innere Medizin (inkl. Pulmologie, Kardiologie), Neurologie, (Neuro‑)Pädiatrie, Geriatrie, Psychiatrie/Psychosomatik, Physiatrie (inklusive Orthopädie, Rheumatologie), Arbeitsmedizin (vgl. [1]).

Die übergeordnete Zielsetzung ergotherapeutischer Interventionen ist die *Stärkung der subjektiven und objektiven Lebensqualität und biopsychosozialen Gesundheit* einschließlich der *Betätigungsgesundheit* [7] der Klientin/des Klienten unter *Berücksichtigung des jeweiligen Lebenskontexts.*

In unserer Versorgungs- und Forschungsarbeit hat sich KRAH®-basiertes Vorgehen [7–11] im Sinne eines evidenzbasierten best-practice-Ansatzes als Mittel der Wahl gezeigt. Responsivität und dialogisches, situations- und kontextsensibles Arbeiten, konsequente Orientierung an den vorhandenen Ressourcen der Betroffenen und ihrer Lebensumwelten, Fokus auf den konkreten Alltag und eine Synthese von top-down und bottom-up sind hierbei im partizipativen ergotherapeutischen Vorgehen handlungsleitend. Die folgenden Ausführungen beziehen sich u. a. auch auf (Praxis-)Evidenzen aus dem PRECISE-Projekt [11, 12].

### Fokus auf individuellen Alltag und damit verbundene Lebensbereiche

Betroffene von Folgen viraler Erkrankung mit SARS-CoV‑2 erleben durch ihre Gesundheitssituation eine Unterbrechung ihrer Handlungsgewohnheiten und Handlungsroutinen, sie sind weniger handlungs- und leistungsfähig („belastungsintolerant“) und können Handlungsrollen mit den damit verbundenen internen wie externen Erwartungen und Aufgaben nicht in gewohnter Qualität und Quantität nachkommen. Dies betrifft ihren Alltag ganz unmittelbar in sämtlichen Lebensbereichen [13]:

- Selbstversorgung (Aktivitäten des täglichen Lebens inklusive Versorgung von Menschen, Tieren, Pflanzen, Wohnumgebung, Finanzen, Kommunikation mit Behörden, …)
- Produktivität (unbezahlte und bezahlte Arbeit inkl. Gefährdung oder Verlust des Arbeitsplatzes, Schule/Aus‑/Weiterbildung/Studium)
- Freizeit
- Erholung (inklusive Schlaf)

Im Setzen *alltagsorientierter Interventionen* gehen Ergotherapeut*innen auf all die für die jeweilige Person relevanten Lebensbereiche, Handlungsfelder, Handlungsrollen und damit verbundenen Aktivitäten, Tätigkeiten und Alltagshandlungen ein.

Ein Betätigungsprofil kann hierbei als hilfreiches Instrument im gesamten ergotherapeutischen Prozess eingesetzt werden und dient u. a. der Selbstbeobachtung, der Identifikation von Problemstellungen und vorhandenen Ressourcen und der (ergotherapeutisch begleiteten) Eröffnung alltagsrelevanter Perspektiven (s. Ausführungen zum ergotherapeutischen Prozess) [11, 14, 15].

## Ausgewählte grundlegende, von Betroffenen als hilfreich rückgemeldete Aspekte in der ergotherapeutischen Arbeit

Im Zuge der biopsychosozialen Gesundheitssituation durch Folgen viraler Erkrankung mit SARS-CoV‑2 sind Betroffene häufig schon nach kurzer Zeit sehr entmutigt, da sie nicht auf ihre gewohnten Fähigkeiten und Fertigkeiten zurückgreifen können und ihren (Lebens‑)Alltag ebenso wie ihre Handlungsidentität – insbesondere in dem Bereich mithilfe ergotherapeutischer Unterstützung – gleichsam „neu erfinden“ müssen [16].

Erste therapeutische Interventionen bedürfen (auch laut problemzentrierter Interviews mit Patient*innen im Rahmen der PRECISE-Studie) u. a. folgender Aspekte, die für eine therapeutische Arbeit grundlegend sind und modellhaft für das weitere soziale Umfeld wirken können:

- einfühlsam und validierend zuhören
- den Schilderungen Glauben schenken, wie die Betroffenen ihre Situation selbst erleben
- die Bemühungen der Betroffenen in Bezug auf die Verbesserung ihrer Gesundheitssituation und Handlungsperformanz im Alltag anerkennen und wertschätzen („an sie glauben“)
- die Betroffenen mit ihren aktuellen Möglichkeiten und Grenzen wahr- und annehmen
- konkrete Perspektiven für das Meistern der gegenwärtigen Situation eröffnen und miteinander erarbeiten
- positive Erfahrungen von Selbstwirksamkeit ermöglichen, Gestaltungs‑, Handlungs- und (wohltuende) Partizipationsmöglichkeiten eröffnen
- Ressourcen auf intra- und interpersoneller Ebene identifizieren und benennen
- vorhandene mentale, emotionale, körperliche, soziale, spirituelle, handlungsbezogene Fähigkeiten wahrnehmen, dem/der Betroffenen bewusst machen und (den ergotherapeutischen Prozess) darauf aufbauen
- Ressourcen im jeweiligen Umfeld identifizieren und im ergotherapeutischen Prozess nutzen, insbesondere im Zusammenhang mit Alltagsgestaltung und Alltagsbewältigung
- konkrete, alltagsrelevante Vereinbarungen für die Zeit bis zum nächsten gemeinsamen Termin miteinander treffen
- ermutigen, die aktuelle Situation als Post-Infektionsphase anzunehmen und schrittweise den Alltag mit professioneller Begleitung wieder neu zu gestalten
- Erreichbarkeit von professioneller Hilfe in Krisensituationen gewährleisten (inklusive diesbezügliche Absprachen mit behandelnden [Ergo-]Therapeut*innen)
- Verlässlichkeit, Bedarfsorientierung und Regelmäßigkeit in der Wahl der Termine bieten (gerade ergotherapeutisches Coaching kann von 2‑mal wöchentlich über 1‑mal/Woche bis zu alle 2 bis 3 Wochen je nach Gesundheits- und Lebenssituation der Betroffenen variieren) [12].

## Einfluss und Bedeutung des therapeutischen Settings

Betroffene von Folgen viraler Erkrankung mit SARS-CoV‑2 unterliegen tageszeitlichen Belastungs- und Leistungsschwankungen, die in der Planung des therapeutischen Settings und bei Interventionen (einschließlich alltagsorientierter ergotherapeutischer Beratung) dringend zu berücksichtigen sind.

Gerade in der *postakuten Phase* können Therapietermine erfahrungsgemäß bevorzugt erst ab dem späteren Vormittag und längstens bis in den späteren Nachmittag hinein für die Betroffenen gesundheitsstärkend wirken. Durch die in der interprofessionellen und interdisziplinären S1-Leitlinie [1] thematisierten Schlafstörungen und bei berichteter und beobachteter Fatigue ist dies besonders bedeutsam. Auch vor und nach (ergo)therapeutischen Interventionseinheiten sind Erholungsphasen wichtig. Konkretes Handeln braucht Energie; im Sinne einer *gesundheitsförderlichen Energiebalance* sind die Wege zur und von der Therapie nach Hause bei der Planung des ergotherapeutischen Prozesses mitzubedenken. Bei Therapie in einer Einrichtung/Praxis ist es oft notwendig, den Patient*innen nach ihrer Anreise eine Ruhephase zu ermöglichen, um anschließend (weitere) therapeutische Interventionen setzen zu können.

Als in der Rehabilitation arbeitende Profession unterstützen Ergotherapeut*innen Betroffene in folgenden Bereichen:

- *stationäre* Rehabilitation (für ältere Menschen: s. auch [17])
- *teilstationäre* Rehabilitation
- *ambulante* Rehabilitation (in entsprechenden Praxen, interprofessionellen Versorgungseinrichtungen, im Hausbesuchssetting, mittels telefonischer sowie teletherapeutischer Beratung und Begleitung)
- im Rahmen der *beruflichen Orientierung und Wiedereingliederung* (vor und während des (stufenweisen) Wiedereinstiegs in Arbeitsprozesse, betreffend bezahlte wie auch unbezahlte Arbeit)
- *sowohl im angestellten als auch selbstständigen Setting*
- im *häuslichen, beruflichen und gemeindenahen Kontext*.

Gezieltere und nachhaltigere alltagsrelevante und alltagsorientierte Interventionen mit unmittelbarer Wirkung u. a. auf Stärkung der Handlungsfähigkeit und Entwicklung von Bewältigungsstrategien sind zumindest in der ersten ambulanten Rehabilitationsphase im *Einzelsetting* möglich.

Ein *Gruppensetting* ist besonders bei von postviraler Fatigue Betroffenen im Hinblick auf Ermutigung für die Bewältigung des Alltags erst in einem bereits deutlich verbesserten Gesundheitszustand hilfreich und effizient.

Fallweise wurden von unseren Patient*innen Wünsche geäußert, auch andere Betroffene von Folgen viraler Erkrankung mit SARS-CoV‑2 kennenzulernen; das angebotene *Gruppensetting* wurde trotz ressourcen- und klientenzentrierter, salutogener Gesprächsführung in den ersten Einheiten von manchen mehr als Belastung denn als Entlastung beschrieben – wegen der Anforderungen, sich auf mehrere Gesprächs‑/Gruppenteilnehmer*innen sensorisch, kognitiv und interaktiv einzulassen, und wegen der psychosozialen Herausforderung im Gruppenkontext: Im Einzelsetting aufgebaute Perspektiven auf die eigene Situation wurden im Vergleich der Verläufe und Situationen anderer nochmals auf die Probe gestellt; zugleich half es gemäß (noch nicht andernorts publizierter) wissenschaftlicher Evaluationsergebnisse, im eigenen Trauer- und Bewältigungsprozess mit professioneller Hilfe weiter zu kommen.

Die Bedürfnisse der Betroffenen sind oft sehr unterschiedlich, ebenso variiert ihre Belastbarkeit; auch die Möglichkeiten, sich auf die Bedürfnisse anderer Personen einzulassen, sind je nach Krankheitsverlauf und -phase eingeschränkt. Entscheidend ist daher das Matching der Gruppenteilnehmer*innen durch die behandelnde Ergotherapeutin/den behandelnden Ergotherapeuten.

Diese Beobachtungen und Erfahrungen legen aus fachlichen ebenso wie gesundheitsökonomischen Gründen nahe, eine Mindestanzahl an Teilnehmer*innen pro Gruppe nicht standardisiert vorzugeben; *inhaltliche Indikationen für die Wahl eines Gruppensettings* sind kritisch zu prüfen, um die gesundheitliche Situation zu stärken und nicht (z. B. durch Überanstrengung und Auslösung von „post-exertional malaise“ [PEMs]) zu schwächen.

Patient*innen im PRECISE-Projekt berichten davon, dass ein sorgsam ausgewähltes Gruppensetting auch Vorteile bieten kann: Der Austausch mit anderen Betroffenen wird insofern als positiv erlebt, als (im Gegensatz zu ihrem Erleben im gesellschaftlichen Kontext) hier keine Rechtfertigung für den aktuellen Zustand erwartet wird. Sie berichten davon, sich von Mitbetroffenen verstanden zu fühlen und den Austausch als wertvoll zu erleben. Voraussetzung dafür ist die Kapazität zur Teilnahme am therapeutischen Angebot inklusive dem konkreten räumlichen Setting.

Gerade für Betroffene von Folgen viraler Erkrankung mit SARS-CoV‑2 ist es wesentlich, den *Ort therapeutischer Interventionen an die aktuelle Gesundheits- und Wohnsituation anzupassen*. Ein *stufenweiser Aufbau* bei Betroffenheit auf der KLOK-Skala [18] von 3 bis 4 könnte folgendermaßen aussehen:

1. *Telefonisches ergotherapeutisches Coaching* (nach kurzen Absprachen zur Terminplanung und Inhalten fürs Selbstmanagement und Coping von je nach Möglichkeit der/des Betroffenen 10 min Dauer weiter steigend)
2. *Tele-Ergotherapie: *Empfehlungen für Telerehabilitation sind bereits international publiziert (vgl. z. B. [19]). Kelsey [20] empfiehlt für die Zeit unmittelbar nach der Entlassung *Follow-ups via Telehealth*. Die Erfahrungen im PRECISE-Projekt haben diese wie folgt weiter präzisiert [11]:
  Teletherapeutische Interventionen sind weiter zu graduieren, indem je nach *Wahrnehmungs- und Verarbeitungskapazität* der Klient*innen mithilfe einer digitalen Plattform (cave: Datenschutz!) die Sitzung auch ohne Nutzen der Kamera‑/Videofunktion erfolgen kann. Dies kann auch alternativ zu telefonischem Coaching genützt werden, da für manche Patient*innen anfänglich auch das Halten eines Endgeräts (Telefonhörers, Handys) (zu) anstrengend ist. Außerdem hilft dieses Setting, die Intimsphäre Betroffener, die sich oft angesichts ihres Bedarfs, auch während einer therapeutischen Sitzung zu liegen, schämen, zu schützen. – Als nächster Schritt kann die Kamera (mit wesentlich mehr sensorischem Input) dazu geschaltet werden. Dies ermöglicht zudem beiderseits das Eingehen auf mimischen Ausdruck. So können auch Übungen angeleitet und therapierelevante Materialien digital geteilt werden. – Bei einigen Klient*innen empfiehlt sich insbesondere angesichts der *geografischen Lage* ein teletherapeutisches Setting: zu viel Energie würden die An- und Rückreise fordern, aufbauende therapeutische Interventionen könnten dadurch weniger greifen. Insofern ist die *Wahl des Settings* unmittelbar abgestimmt mit dem jeweils notwendigen *Haushalten mit der verfügbaren Energie* der betroffenen Person.
3. *Präsenz: *Auch hier gilt es, *stufenweise* vorzugehen. Für Klient*in, Therapeut*in wie auch Zuweiser*in ist zu bedenken, dass der Weg zur und von der Therapie bereits Energie erfordert – Energie für die (Handlungs- und motorische) Planung dieser Unternehmung einschließlich der Organisation von Verkehrsmitteln (Autofahren ist meist in postakuter Phase nicht möglich; auch das Benützen öffentlicher Verkehrsmittel stellt für viele eine in dieser Zeit unüberbrückbare Barriere dar – weitere Gründe, die für ein teletherapeutisches Setting sprechen können). Nach Arzt- und Therapieterminen schildern Betroffene „Zusammenbrüche“ ihrer Energie noch am selben Tag oder aber unmittelbar darauf in den Folgetagen. An einem „Tag mit Arzt- und/oder Therapietermin außer Haus“ können die Klient*innen je nach Betroffenheit keine sonstigen Tätigkeiten einplanen. In dieser Phase ist es besonders wichtig, die (Präsenz-)Termine umgehend zu stoppen, wenn Müdigkeit beobachtet wird. Sollte im Sitzen gearbeitet werden, ist die Verfügbarkeit einer Therapieliege zum Ausruhen in solchen Situationen sehr empfehlenswert.
  In unserer Praxisforschungserfahrung haben sich erste Ergotherapietermine im Ausmaß von 10–15 min als ausreichend anspruchsvoll und effizient erwiesen; eine Therapieeinheit im Umfang von 45–60 min wahrnehmen zu können gilt bereits als großer Erfolg im Behandlungsverlauf.
  Auch bei der *Gestaltung des Therapieraumes* ist dringend darauf zu achten, dass es möglichst natürliche Lichtquellen gibt; künstliches Licht sollte indirekt beleuchten. Viele unserer schwerer betroffenen Patient*innen wünschen sich vorerst einen möglichst dunklen Raum (auch im Sommer). Auch andere *sensorische Reize* sind zu beachten: Viele der Betroffenen reagieren sehr sensibel auf Gerüche; in dem Raum sollte nach Möglichkeit unmittelbar davor nicht gekocht oder gegessen werden. Neutrale Geruchsumgebung und natürliche ätherische Öle kommen hingegen in der Regel sehr gut an. Das Tragen einer FFP2-Maske ist (war) für Patient*innen, die Mühen beim Atmen und im Hinblick auf ihre Sauerstoffsättigung haben, herausfordernd. Auch akustische Reize sollten nach Möglichkeit wohlüberlegt sein; schalldichte Fenster sind für therapeutische Räumlichkeiten für diese Patient*innen notwendig. Selbst das Geräusch beim Abreißen eines Papiers, das Vorbeifahren eines Verkehrsmittels, das Öffnen einer Türe oder Lade kann sie durch die einwirkenden sensorischen Reize belasten. Auch die Tageszeit für die Therapie sollte sich nach den Aufnahme- und Verarbeitungskapazitäten der Betroffenen richten.

Eine weitere Setting-Möglichkeit sind Online-Kurse, die das Selbstmanagement der Betroffenen stärken. Erfahrungen zu einem multiprofessionellen online-Angebot dokumentieren beispielsweise Flannery et al. [21]; Zugänglichkeit und *gesundheitliche Chancengerechtigkeit* müssen bei der Wahl des therapeutischen und Versorgungs-Settings berücksichtigt werden.

Bei der *Wahl und Gestaltung des (therapeutischen) Settings* müssen unterschiedliche Aspekte in Betracht gezogen werden, die je nach (Gesundheits‑)Situation der Klient*innen anzupassen sind. Das jeweilige therapeutische Setting ist als Übungssituation für ein selbstständiges Meistern des jeweiligen Alltags, der jeweiligen gesundheits- und handlungsbezogenen Herausforderungen zu verstehen. Bei der Planung der Dauer einer Interventionseinheit sollen auch An- und Abreisedauer zur Therapie berücksichtigt werden. Diese können für die Klient*innen, wie oben beschrieben, eine erhebliche Belastung darstellen und somit die Dauer der Einheit deutlich verkürzen.

Wesentlich für die (ergo)therapeutische Begleitung sind Kontinuität, Verlässlichkeit und Abstimmung der Frequenz und Dauer der Therapieeinheiten auf die gesundheitliche Situation der Betroffenen. Hier empfiehlt sich für Kostenträger im Sinne der Effizienz und Effektivität, Pauschalen für ergotherapeutische Versorgung zu bewilligen, die individuell dem jeweils aktuellen (da stark fluktuierenden) Gesundheitszustand angepasst genutzt werden können.

Ergotherapie sollte auch in Österreich als Angebot in die Regelversorgung von Menschen mit Folgen viraler Erkrankung mit SARS-CoV-2 in sämtlichen Settings aufgenommen werden. Die Finanzierung therapeutischer Leistungen dient u. a. auch gesundheitlicher Chancengerechtigkeit, zumal die finanzielle Situation der Betroffenen durch Langzeitkrankenstände teilweise sehr angespannt ist.

Den aktuell verfügbaren Praxiserfahrungen zufolge umfassen notwendige Behandlungsdauern pro Jahr pro Patient*in (in Stunden ausgedrückt) 30 bis 50 Einheiten Ergotherapie.

## Ergotherapie-Indikation in den verschiedenen Prozessphasen der Versorgung von Betroffenen mit Folgen viraler Erkrankung mit SARS-CoV-2

In der internationalen Literatur wird die Bedeutung von Ergotherapie im Zuge des Entlassungsmanagements hervorgehoben [3]. Dabei geht es insbesondere um Beratung und Coaching der Betroffenen im Hinblick auf den Transfer in den häuslichen Alltag unter Berücksichtigung von:

- Körperfunktionen (Atmung, körperliche Belastungsfähigkeit, kognitive Leistungsfähigkeit, Wahrnehmungsverarbeitung [Verarbeitung sensorischer Reize aus der Umwelt], Schmerzen, Schlaf, emotionale Situation, vegetatives Nervensystem und seine aktuellen Wirkweisen)
- Aktivitäten (Was ist bei Entlassung im stationären Setting möglich zu tun? Was kann unter welchen Bedingungen in den jeweiligen Lebensalltag zum gegenwärtigen Zeitpunkt transferiert werden? Tägliches und wöchentliches Planen und Priorisieren, langsames Erhöhen des Energielevels) [3, 15, 22]
- Partizipation (Welche Handlungsrollen sind im jeweiligen Alltag bedeutsam? Welchen kann wie nachgegangen werden?)
- Umweltbezogene Kontextfaktoren (Welche Art der Unterstützung ist im jeweiligen Lebenskontext des/der Betroffenen vorhanden? Wer braucht welche Art der Information im Hinblick z. B. auf Pacing, Vermeidung von „post-exertional malaise“? Welche Umweltadaptierung ist für die kommenden Tage/Wochen notwendig? – z. B. Information an Arbeitgeber*innen, bedarfsorientierte Organisation von Hilfsmitteln, Beratung hinsichtlich sensorischer und handlungsrelevanter Aspekte der Wohnumgebung wie mögliche Verdunkelung der Räume bei Lichtempfindlichkeit, Schutz vor Lärm/Geräuschen u. a. m.; Nahtstellenmanagement im Sinne der Kooperation, Information, Vernetzung, Empfehlung nachfolgender Ergotherapie und anderer relevanter Berufsgruppen)
- Personbezogene Kontextfaktoren (Stärkung der Gesundheitskompetenz, der Resilienz und des Copings beispielsweise durch Mitgeben von Informationsunterlagen zu Pacing, Selbstmanagement, Empfehlung eines Betätigungsprofils/Aktivitätenprotokolls)
  - zur ressourcenorientierten Selbstbeobachtung
  - für Energie- und bedarfsweise Fatiguemanagement und
  - als Unterstützung in gesundheitsförderlicher Tages- und Wochengestaltung
  - zur gemeinsamen Evaluation des bisherigen Genesungsverlaufs mit Aussicht auf die konkreten nächsten Schritte [12, 23].

Die hier im Zuge des *Entlassungsmanagements* genannten Ansatzpunkte betreffen auch den weiteren ambulanten Versorgungsprozess, wobei hier vorerst bedarfsorientiert vereinbarte Termine (s. oben) im *Einzelsetting* zu empfehlen sind. Inhaltliche Orientierung gibt dabei KRAH®-basiertes Arbeiten mit Fokus auf die Anliegen und Ressourcen der jeweiligen Person, ihrer Lebensumwelt und der potenziell freudvollen, aktuell möglichen und zukünftig erstrebenswerten Handlungen für einen Alltag mit höchstmöglicher Lebensqualität [8, 9, 11].

Ergotherapie findet in dieser Versorgungsphase je nach Sinnhaftigkeit und Möglichkeit auch *im häuslichen Setting* statt, um unmittelbar auf die alltägliche Situation eingehen zu können.

*In Praxen und anderen Versorgungseinrichtungen* umfassen ergotherapeutische Interventionen u. a. auch kognitive, wahrnehmungs- und körperzentrierte sowie ausdrucks- und kompetenzzentrierte Methoden vorbereitend für bzw. in Verbindung mit alltagsorientierten Interventionen (u. a. Planung und Ausprobieren von Alltagsaktivitäten, Energiemanagement, Beratung/Edukation zu Hilfsmitteln), in direkter Antwort auf die momentanen Bedürfnisse und Notwendigkeiten für den betroffenen Patienten/die betroffene Patientin.

Anfangs kann im Rahmen der Ergotherapie mit Betroffenen von Folgen viraler Erkrankung mit SARS-CoV‑2 *salutogenes und Resilienz stärkendes, alltagsorientiertes ergotherapeutisches Coaching* im Vordergrund stehen [8, 9, 11, 24]; abgesehen von beispielsweise *Impulsen zum Fokus auf das, was gerade gelingt, was aktuell guttut, was in den vergangenen Tagen wohltuend war und worauf sich die/der Betreffende in den nächsten Tagen freut*, können *körper- und wahrnehmungsorientierte Angebote zur positiven Regulation des parasympathischen Nervensystems* sowie *mental stärkende Tätigkeiten* Mittel der Wahl sein. Auch hier gilt es, das aktuell Wesentlichste zu filtern und unmittelbar auf eine Überforderung durch die Patientin/den Patienten zu reagieren.

*Stationäre Rehabilitation* hat den Vorteil, für die Betroffenen von Folgen viraler Erkrankung mit SARS-CoV‑2 hochfrequenter und kompakt therapeutische Unterstützung anzubieten [17]; gleichzeitig kann dies aber auch leicht zu Überforderung und Rückfällen (PEMs, „Crashes“) führen, insbesondere wenn die Einhaltung einer gewissen Anzahl und Dauer von Interventionseinheiten ohne Möglichkeit, auf die jeweilige Situation einzugehen, gefordert ist. Durch die *Überempfindlichkeit sensorischen Reizen* gegenüber (z. B. sich bewegenden Menschen im gleichen Raum, raschem Wechsel visueller Eindrücke, Geräuschpegel) kann sowohl eine stationäre Rehabilitation als auch ein Gruppenangebot im ambulanten Setting zusätzliche Belastungen mit sich bringen. Insofern ist *dringend darauf zu achten, welches Angebot zu welchem Zeitpunkt für wen indiziert ist.*

Nachdem sich auch die *Ernährung* unmittelbar auf die Energie und Handlungsfähigkeit auswirkt und gerade diese Patient*innengruppe darauf sehr sensibel reagiert, ist die Qualität der Nahrung auch im stationären rehabilitativen Setting für einen Erfolg der multiprofessionellen Bemühungen essenziell.

Ähnliches gilt auch für das häusliche Umfeld: bei entsprechender medizinischer Unterstützung z. B. durch gezielte Infusionstherapie, durch das Einhalten der relevanten ernährungsmedizinischen Empfehlungen für Folgen viraler Erkrankung mit SARS-CoV‑2 in Abstimmung mit dem subjektiven Wohlbefinden können u. a. ergotherapeutische Hilfestellungen besser greifen und in den Alltag nachhaltiger transferiert werden.

Der Aufbau der Ergotherapie ist individualisiert (personzentriert) an den jeweils vorliegenden Möglichkeiten und Grenzen, Handlungsbedürfnissen und Notwendigkeiten der Patient*innen orientiert und erfolgt stufenweise und konsequent ressourcenorientiert, ohne Probleme zu negieren. Dabei ist eine Kombination eines Top-down- UND Bottom-up-Ansatzes notwendig, wie beispielsweise im KRAH®-Ansatz [7, 10] beschrieben und in ergotherapeutischen Modellen ebenso wie in der ICF [25] verankert.

Teil der voraussetzenden Arbeit mit den Betroffenen im Hinblick auf eine Wiederaufnahme ihrer bezahlten und/oder unbezahlten Arbeit ist die *Stärkung einer gesundheitsförderlichen Betätigungsbalance im jeweiligen Alltag.* Wenn die Lebensbereiche Selbstversorgung, Erholung (einschließlich Schlaf, Einhalten von und Regenerieren in Pausen) und selbstgestaltete, freie Zeit wieder selbstorganisiert gut erlebbar sind, können auch andere Verpflichtungen und Routinen wie Erwerbsarbeit stufenweise wiederaufgenommen werden [11, 26].

Jede dieser Transitionen erfordert eine Anpassung von Handlungsroutinen und häufig auch den Aufbau neuer Handlungsmuster mit Auswirkungen auf die eigene Handlungsidentität, auf Handlungsrollen und auf das jeweilige soziale Umfeld.

## Weitere Empfehlungen für die ergotherapeutische Arbeit mit Betroffenen von Folgen viraler Erkrankung mit SARS-CoV-2, mit Orientierung am ergotherapeutischen Prozess

In der Arbeit mit Betroffenen von Folgen viraler Erkrankung mit SARS-CoV‑2 sind einige grundlegende Aspekte für alle Altersgruppen wesentlich für eine Intervention, die für die Einzelnen hilfreich sein kann:

- responsives, situations- und kontextsensibles Arbeiten
- wahrnehmen nonverbaler Cues (inklusive Wechseln der Gesichtsfarbe, Abschweifen des Blicks, Erhöhung der Herzfrequenz, sichtliches Ermüden) und unmittelbares Darauf-Eingehen (z. B. in Form von Pause ermöglichen, Reizreduktion, Herabsetzen der Anforderung, evtl. auch vorzeitiges Beenden der Therapieeinheit)
- Phasen VOR einer Belastungsgrenze wahr- und ernst nehmen lernen (dies zunächst den Patient*innen selbst vermitteln, mit ihnen erproben), inklusive Graduieren von Tätigkeiten und Anpassen von Umwelten
- komplexes Fachwissen aus verschiedenen, für die Erkrankung mit SARS-CoV‑2 relevanten Fachbereichen (wie u. a. Neurologie, Psychosomatik, Innere Medizin, Neuropädiatrie, Physiatrie) für optimale Behandlung/Beratung/Coaching einer Klientin/eines Klienten notwendig (vgl. [1])
- Kombination und flexibles Nutzen einer Synthese und Integration von Top-down- und Bottom-up-Vorgehen [7, 10, 11].

### Erstkontakt

Wichtig dabei ist aktives Zuhören, Abholen bisheriger Befunde, ressourcenorientierte, validierende Gesprächsführung, Identifikation von Ressourcen der/des Betroffenen selbst und deren sozialer, physischer und sonstiger Umwelt. Wesentlich ist zudem, Anliegen und Auftrag der Klientin/des Klienten zu klären und mit den Möglichkeiten alltagsorientierter, handlungsorientierter Ergotherapie abzustimmen. Auch die Abklärung von aktuellen Belastungsmöglichkeiten und -grenzen zur gemeinsamen Auswahl des gegenwärtig passenden therapeutischen Settings ist wichtig.

Weitere involvierte Berufsgruppen sollten ebenso erhoben und Wunsch zur Netzwerkarbeit seitens des/der Betroffenen abgeklärt werden.

Es empfiehlt sich, bereits beim Erstkontakt eine Empfehlung z. B. zur Erstellung eines Betätigungsprofils bzw. Aktivitätenprotokolls mit Festhalten wohltuender Momente bis zur nächsten Ergotherapie-Einheit mitzugeben.

#### Befunderhebung

Bei der Befunderhebung ist bereits bei der Auswahl geeigneter Assessments und Tools die derzeitige Belastungssituation der Patient*innen zu berücksichtigen. Dies bezieht sich sowohl auf *Art und Umfang als auch auf den Inhalt der Assessments*.

RCOT [3] empfiehlt, im Sinne des Energiemanagements die Durchführung von (auch standardisierten) Assessments bei Bedarf in mehrere Teile und möglicherweise auf mehrere Termine aufzuteilen.

Eine *ICF-basierte Befunderhebung und Dokumentation* bietet die Möglichkeit, die komplexen Situationen und Wechselwirkungen zwischen der Gesundheitssituation der Betroffenen, der Körperstrukturen und -funktionen, Auswirkungen auf Aktivitäten und Partizipation, den Einfluss von personbezogenen und Umweltfaktoren zu erfassen und gezielt auf spezifische Aspekte einzugehen.

Viele Menschen mit Folgen einer viralen Erkrankung mit SARS-CoV‑2 können zumindest vorerst ihrer beruflichen Tätigkeit nicht nachgehen. Finanzielle und existenzielle Themen im Zusammenhang mit *Armutsgefährdung bzw. Armutsbetroffenheit* sind gleich zu Beginn der ergotherapeutischen Arbeit im interprofessionellen Netzwerk zu erheben; durch person- und umweltbezogene Maßnahmen können diese präventiv ergotherapeutisch adressiert werden [12].

Mithilfe eines *Betätigungsprofils* können der aktuelle Alltag und relevante Handlungsrollen (bisherige wie gegenwärtige und wünschenswerte) der Klient*innen erfasst werden [12]. Beim gemeinsamen Ausfüllen z. B. eines „normalen“/beispielhaften Tages, des vorangegangenen Tages oder der letzten oder aktuellen Woche kann ein Überblick über bereits vorhandene Ressourcen und mögliche Problemstellungen im Alltag der Klient*innen entstehen. Auf dessen Basis können weitere relevante, spezifische (ergotherapeutische) Assessments ausgewählt werden.

Ethische und fachliche Dilemmata entstehen (auch interprofessionell) bei der Durchführung normreferenzierter Assessments, da diese einerseits die stark reduzierten Energiekapazitäten der Betroffenen (über)beanspruchen können und gleichzeitig oft nicht über hier zutreffende Normwerte verfügen. Auch Kolleg*innen aus dem Bereich der Neuropsychologie berichteten davon, auf standardisierte, umfangreiche Assessmentprozesse bei diesem Klientel vorerst zugunsten von stützender Gesprächsführung aus fachlichen und ethischen Gründen zu verzichten.

#### Ausgewählte mögliche Assessment- und Interventionsinstrumente in der Ergotherapie

Folgende Assessments können im Rahmen der Ergotherapie mit Betroffenen von Folgen viraler Erkrankung mit SARS-CoV‑2 eingesetzt werden:

*Handlungsperformanz und Handlungsrollen*

- Canadian Occupational Performance Measure (COPM) [27]
- Occupational Performance History Interview (OPHI-II) [28]
- Occupational Questionnaire [29]
- Occupational Self Assessment (OSA) [30]
- Rollencheckliste [15, 29]

*Handlungsinteressen*

- Betätigungsprofil [12]
- Interessenscheckliste [29, 31]

*Energiemanagement bzw. Fatigue-Management *[22]

- Fatigue Severity Scale (FSS) [32]
- Fatigue Assessment Scale (FAS) [33]
- Skala der Selbstwirksamkeitserwartung in der Benutzung energiesparender Strategien (SWE-BESS) [34]
- Time-Use-Tagebücher/Betätigungsprofil/Energievase (z. B. mithilfe von Apps) [35]

*Lebensqualität*

- Kidscreen [36]
- SF-36 [37]
- WHOQOL-BREF [38]

*Diagnosespezifisch*

- Post-COVID-19-Functional-Status Scale [18]
- Covid-19 Yorkshire Rehabilitation Screening (C19-YRS) [39, 40]

*Assessments für spezifische Körperfunktionen*

- Subjektives Belastungsempfinden: BORG-Skala [41]
- Körperliche Ausdauer/Belastbarkeit: „1-minute-sit-to-stand-test“ [15, 42];
- Handkraftmessung mit Dynamometer [43, 44]
- Montreal-Cognitive-Assessment-Test (MoCa): zur Erhebung der kognitiven Leistungsfähigkeit [45].

#### Zielfindung und Zielsetzung

Die partizipative und kontextbezogene Zielfindung und Zielsetzung (vgl. [7, 10, 15]) erfordert in der ergotherapeutischen Arbeit mit Betroffenen von Folgen viraler Erkrankung mit SARS-CoV‑2 viel Gespür und situative Anpassung je nach aktueller biopsychosozialer Gesundheitssituation der Patient*innen. Kurzfristige, unmittelbare Ziele, die die Resilienz und das Meistern des Alltags stärken und positive Erfahrungen der eigenen Wirksamkeit und Gestaltungskraft eröffnen, stehen mit langfristigen Zielen in für die Patient*innen greifbarer Abstimmung. Letztere betreffen langfristige Partizipationsanliegen, wie z. B. den Wiedereinstieg ins Arbeitsleben. In jedem Fall gilt auch hier, den jeweiligen Lebenskontext in die Zielsetzung gedanklich oder auch konkret einzubeziehen.

Auch die COAST-Methode stellt eine Möglichkeit zur handlungsorientierten und Ergotherapie-spezifischen Zielformulierung dar. „Werden Ziele präzise bestimmt, stärkt dies die Arbeitsbeziehung zwischen Klient*innen und Therapeut*innen und hat positive Effekte im Hinblick auf funktionelle Verbesserungen, Performanz, einen unterstützenden Einfluss auf die Selbstwirksamkeit und das Gefühl des Eingebundenseins in den Therapieprozess.“ [46]

Im Prozess der Zielfindung und Zielsetzung gilt es zu beachten und ggf. sowohl den Patient*innen als auch Angehörigen, Kostenträgern und anderen relevanten Berufsgruppen zu kommunizieren, dass nicht nur die zeitliche Festlegung zur Erreichung des Ziels, sondern auch die Zielsetzung selbst im Verlauf prozessual evaluiert und in Abhängigkeit von eventuell auftretenden PEMS oder „Crashes“ bedarfsorientiert angepasst werden muss [11, 47].

#### Interventionen planen und setzen

##### Ansatzpunkt PERSON

Grundsätzlich stärken Ergotherapeut*innen Menschen mit gesundheitsbezogenen Problemstellungen in ihren Gesundheits- und Lebenskompetenzen durch klientenzentriert und alltagsorientiert relevante Informationen und die partizipative Erarbeitung entsprechender Fertigkeiten (Skills). Dies gilt auch für Symptome, die postviral nach Erkrankung mit SARS-CoV‑2 beobachtet werden können [35, 48].

Die Situation von Betroffenen von Folgen viraler Erkrankung mit SARS-CoV‑2 erfordert nicht weniger als eine (Neu‑)Betrachtung des Umgangs mit dem Leben, mit sich selbst [3, 11].

Im Rahmen der Ergotherapie können Klient*innen dahingehend begleitet werden, ihre neue (temporäre) (Handlungs‑)Identität sowie Veränderungen der Handlungsrollen bzw. der Qualität und Quantität und deren Lebbarkeit in der gegenwärtigen Phase anzunehmen [3].

*Im Umgang mit den jeweiligen Gesundheitsproblemen sind sowohl Selbst- als auch Co-Management* bedeutsam [3, 15, 19, 22, 49]. Letzteres erfordert das Annehmen und Fragen um Hilfe, was gerade in unserem Kulturkreis für viele neu zu erlernen ist.

Im Rahmen der Ergotherapie wird die Handlungsbiografie der jeweiligen Person beachtet. Ressourcenorientierte Biografiearbeit baut am Handlungspotenzial auf und eröffnet im dialogischen, partizipativen Arbeiten neue Perspektiven für das eigene Leben.

In Ergänzung zu körperbezogenen Interventionen gilt es aus ergotherapeutischer Sicht, die Betroffenen auch insbesondere in ihrer *psychosozialen Gesundheit* zu stärken.

Dies betrifft u. a.: die Haltung und Einstellung zu sich selbst (sich selbst auch in dieser Situation annehmen, die Situation annehmen, sich selbst und eigene Bedürfnisse wahr- und ernst nehmen), sowie zu ihrer aktuellen Gesundheits- und Lebenssituation, insbesondere im Hinblick auf Veränderungen in Handlungsrollen, Handlungsidentität, Handlungsroutinen; die Stärkung ihres Copings im Hinblick auf Alltagsbewältigung und Alltagsgestaltung; das Eröffnen von Perspektiven der Zuversicht im Hinblick auf Gestaltungsmöglichkeiten des jeweiligen Alltags.

Handlungsorientierte ergotherapeutische Interventionen für die jeweilige Person stärken die Selbstwirksamkeit bei der Bewältigung von Herausforderungen im Alltag und begleiten in notwendigen Verhaltensänderungen, die die Resilienz der Betroffenen stärken. Diese Kompetenzen des Selbstmanagements drücken sich u. a. im handlungsbezogenen Pacing [22, 50] aus.

##### Energiemanagement/Fatigue-Management (s. auch S1-Leitlinie zu Fatigue im Rahmen von Folgen viraler Erkrankung mit SARS-CoV-2)

Ein zentrales Thema für Betroffene von Folgen viraler Erkrankung mit SARS-CoV‑2 ist Energie, der Umgang mit reduzierten Energiereserven, der eigene Energiehaushalt, wie und wofür die vorhandene Energie eingesetzt wird/werden kann [50–52]. Betroffene erleben eine Veränderung der Belastung durch Handlungen und Situationen, die früher nicht belastend waren (z. B. kochen bzw. Essen zubereiten, duschen, Haare waschen, einkaufen, u. v. m.). Viele berichten von einem Bedürfnis und der Notwendigkeit von Ruhe, ruhiger Umgebung und vielen Pausen.

Bewusst *Pausen* einzubauen, deren notwendigen und wirksamen Zeitpunkt sowie deren Länge und Qualität zu identifizieren ist somit Teil der Ergotherapie *vom akuten Setting bis hin zur Wiedereingliederung in Arbeitsprozesse *[3, 15, 22, 53, 54].

Als Teil ergotherapeutischen Coachings, ergotherapeutischer Beratung und Begleitung sind

- *kognitive Aspekte* [55, 56]
- sensorische Aspekte [11]
- *(Ein- und Durch‑)Schlaf(-qualität und -dauer)* [55, 57]

auch im Hinblick auf von sich selbst und seitens der Umwelt erwartete Aktivitäten und Handlungen zu berücksichtigen.

Ziel ist es, eine möglichst *gesundheitsförderliche Betätigungsbalance im Rahmen der aktuellen Handlungs- und Partizipationsmöglichkeiten* zu eröffnen [15, 22]. Dies bedarf eines integrativen Verständnisses von Fatigue im Kontext von Folgen viraler Erkrankung mit SARS-CoV‑2 [1].

Ergotherapeut*innen stärken Klient*innen in ihren Coping-Strategien und in ihrer Resilienz; dabei kann Achtsamkeitspraxis als evidenzbasierter Schutz‑/Resilienzfaktor im Rahmen alltagsorientierter Ergotherapie mit adressiert und im Rahmen therapeutischer Interventionen erprobt werden [55, 58, 59].

Zu den ergotherapeutischen Kernkompetenzen zählen das Analysieren und Graduieren von Aktivitäten und Tätigkeiten mit Berücksichtigung der personalen und umweltbezogenen Kontextfaktoren, was auch im Energiemanagement für die Betroffenen eine große Hilfe darstellen kann [22, 55, 60].

##### Implementierung von Pacing als Strategie im Alltag – Vermeidung von PEMs

Eine auch in der internationalen Literatur als bewährt geltende Methode zur Vermeidung von PEMs ist das *Pacing*. Die Kunst besteht u. a. darin, stets (etwas) weniger zu tun, als es die Kraft gegenwärtig erlauben würde [61].

Ergänzend zu den vom Altea Netzwerk [6] empfohlenen Handlungsstrategien hat sich in der ergotherapeutischen Forschung im PRECISE-Projekt gezeigt, dass das Pacing im Hinblick gerade auf das, *was wo wie wann wie lange mit wem getan wird*, anzuwenden ist (vgl. [11, 20]).

Wesentliche Aufgabe für die Betroffenen mit Fatigue und Belastungsintoleranz („post-exertional malaise“ [PEM]) und das Behandlungsteam ist es, *Strategien zur Vermeidung von belastungsassoziierten Verschlechterungsphasen,* sog. „Crashes“, zu entwickeln.

Voraussetzung für die Vermeidung von „Crashes“ ist das frühzeitige *Wahrnehmen bzw. Erkennen von eigenen Grenzen* und deren „Prodromalphase“. Auch die *Akzeptanz* aktueller Einschränkung(en) durch den/die Betroffene/n selbst sowie das jeweilige soziale (inklusive professionelle) Umfeld ist Inhalt der ergotherapeutischen Arbeit und bedingt diese mit.

Beide Prozesse brauchen je nach Lebenssituation und Handlungsbiografie sowie Persönlichkeit der Betroffenen unterschiedlich lange Zeit und bedeuten oftmals essenzielle Auseinandersetzung mit der eigenen Handlungsidentität, mit vergangener, gegenwärtig möglicher und wünschenswerter Art der Ausfüllung von Handlungsrollen, mit eigenen und fremden Erwartungen und Ansprüchen an die eigene Handlungs- und Leistungsfähigkeit im Alltag.

Auch die *Berücksichtigung sensorischer Reizschwellen*, die bei vielen Betroffenen durch die Erkrankung, häufig passager, verändert und deutlich niedriger sind, ist wesentlich für die Vermeidung belastungsinduzierter Rückschläge. Dies betrifft olfaktorische, gustatorische, visuelle, auditive, taktile und vestibuläre Reize ebenso wie die Temperaturempfindung (zu heiß und zu kalt).

Ergotherapeut*innen nutzen in Beratung und Coaching der Betroffenen *Handlungsperformanzanalysen* (einschließlich Umwelt‑, Aktivitäts‑, Anforderungs- und Fähigkeitsanalysen), um passende Voraussetzungen für die Durchführung von Tätigkeiten gemeinsam mit den Betroffenen auswählen und gestalten zu können.

Anders als gesellschaftlich und in der Trainingstherapie üblich, bedeutet ein (Wieder‑)Aufbau von Fähigkeiten inklusive Leistungsfähigkeit hier also NICHT, an bzw. über die eigenen Grenzen zu gehen, sondern diese vorerst anzuerkennen und innerhalb der Möglichkeiten aktiv zu sein.

Zudem sind *Strategien zur Entspannung, Regeneration und zur Erholung (inklusive Schlaf)* zu vermitteln bzw. seitens der Betroffenen zu erlernen/zu entwickeln [12, 55, 57, 62, 63].

Dabei können unterschiedliche Möglichkeiten zur Terminisierung und Gestaltung von Pausen, Erholungs- und Regenerationsphasen im Hinblick auf den konkreten Lebensalltag der Betroffenen im Rahmen ergotherapeutischer Intervention (Beratung, Coaching, Therapie) gemeinsam ausgewählt und mit professioneller Begleitung im konkreten Alltag erprobt werden.

Unsicherheit über die weitere Entwicklung ihrer Gesundheitssituation und deren Auswirkungen auf den Lebensalltag sowie die Veränderungen im Hinblick auf Handlungsgewohnheiten, Handlungsroutinen, Handlungsrollen, Handlungsidentität fordern die Klient*innen in sämtlichen Lebensbereichen [12, 64] und betreffen auch den der Erholung.

Klient*innen trauern oft darüber, bisherige für sie wertvolle Tätigkeiten, die sie als energiebringend und erholsam erlebten, nicht mehr durchführen zu können. Hier ist es auch eine Aufgabe der Ergotherapie, diesen Prozess anzuerkennen und gemeinsam mit den Betroffenen nach Möglichkeiten für Coping und Adaptierung zu suchen; dies auch gerade dann, wenn die Bereitschaft zum Ersetzen der bisher vertrauten durch andere Tätigkeiten (z. B. zur Erholung) (noch) nicht gegeben ist. Gerade im Erleben der eigenen Defizite mit Auswirkungen auf Handlungs- und Partizipationsmöglichkeiten ist es laut Praxisevidenz wesentlich, das bereits Gelingende zu benennen bzw. sichtbar zu machen [12]. Dies kann alleine schon das Wahrnehmen des vereinbarten Termins und das weiterhin „Dran-Bleiben“ betreffen.

Der Einsatz des Betätigungsprofils als ergotherapeutisches Prozessinstrument hat sich mit Betroffenen von Folgen viraler Erkrankung mit SARS-CoV‑2 im ambulanten Setting bewährt [12]. Die niederländischen Ergotherapie-Leitlinien für „post COVID“ enthalten die Empfehlung des Mini-Aktivitäten-Ansatzes [65], das laut österreichischen Ergotherapeut*innen mit diesem Klientel mit entsprechender klientenzentrierter Adaptierung anzuwenden ist [5]^1^.

### Erholung und Schlaf (vgl. auch [62])

Klientenzentriert und personalisiert können hier u. a. folgende Ansatzpunkte, Methoden, Maßnahmen und Mittel in der Ergotherapie im interprofessionellen Kontext eingesetzt werden:

- Schlaftagebücher, Fragebögen zu Schlaf [55, 57]
- Aktuellen Biorhythmus identifizieren – notwendige Erholungsphasen (z. B. mithilfe eines Betätigungsprofils) erheben (auch Pacing dabei berücksichtigen) [12]
- Orientierung an Tageszeiten, die zumeist mit mehr Energiekapazität erlebt werden, für die Durchführung von Tätigkeiten (Betätigungsbalance)
- Analyse der Handlungsroutinen und Handlungsgewohnheiten sowie der sensorischen Reize in der Zeit vor dem Schlafengehen [12, 57, 63]
- Erarbeitung bzw. Adaption von Ritualen zur Erholung oder vor dem Schlafengehen
- Beratung zur Gestaltung von Umwelten, in denen Erholung und Schlaf stattfinden – Schlafhygiene
- Kognitive Verhaltenstherapie bei Insomnie (CBT-I), Umweltanpassungen, Lichttherapie [57, 63]
- Lifestyle-Management, Betätigungsbalance [55].

### Kognitive Hilfestellungen

Angesichts der neuropsychologischen Problemstellungen insbesondere im Bereich der *Handlungsplanung (Sequenzierung), motorischen Planung, praktischen Problemlösefähigkeit, Reizselektion/Priorisierung, Informationsverarbeitung Konzentration, Gedächtnisleistungen, Wortfindung/Sprache* [56, 66–69] hat sich in der wissenschaftlich begleiteten ergotherapeutischen Versorgungspraxis gezeigt, dass ein unmittelbares KRAH®-basiertes Vorgehen [7–10] bessere Wirkung zeigt als das Durchführen standardisierter Interventionen (dies betrifft auch den nach ethischen und professionellen best-Practice-Kriterien abzuwägenden Einsatz standardisierter und normierter Assessment-Tools, wie im Rahmen der Befunderhebung angeführt) [23].

Auf die jeweils vorhandenen neuropsychologischen Problemstellungen eingehend, sind *ergotherapeutisch individualisierte, alltagsorientierte Hilfestellungen* anzubieten. Dies betrifft die Empfehlung und Beratung hinsichtlich sensorisch leichter handhabbarer *Umweltaspekte* wie auch Coaching im Hinblick auf *alltagsorientiertes Selbstmanagement* [4].

Neurokognitives Training hat sich im Rahmen unserer Arbeit dann als sinnvoll erwiesen, wenn *unmittelbare Alltagshandlungen* therapeutisches Mittel und Ziel waren. Zu Beginn betraf dies alleine schon das Einhalten des vereinbarten Termins, die notwendige Organisation dafür, die Konzentration während des Gesprächs, das aufmerksame Zuhören und Aufnehmen verbaler und visueller Informationen, das Filtern von Störgeräuschen oder einfallendem (z. B. Sonnen‑)Licht, das Erinnern und Mitteilen alltagsrelevanter Anliegen an die gemeinsame Arbeit.

„Traditionelles“, gerade im Bereich der neurologischen Rehabilitation übliches kognitives Training raubt laut Praxisevidenzen bei Betroffenen von Folgen viraler Erkrankung mit SARS-CoV‑2 mit Betroffenheit von Grad 2, 3 und 4 auf der KLOK-Skala oft mehr Energie, als es Nutzen und Erfolg bringt [12, 68]; je nach Situation ist dieses daher insofern zu adaptieren, als persönlich relevante, alltagsorientierte Inhalte adressiert werden; dies betrifft auch den „Reha-Alltag“ in stationärer wie ambulanter Rehabilitation.

Ergotherapie mit u. a. alltagsorientiertem kognitivem Training zur Vermittlung und Erarbeitung von *Handlungsplanung, Gedächtnisstrategien und -hilfen *kann (bevorzugt) im Einzel-, fallweise auch im Kleingruppensetting angeboten werden; ideal ist dabei, diese schon im Rahmen der Therapie zur Lösung der konkreten Alltagsproblematik auszuprobieren und den Transfer in den jeweiligen Alltag zu begleiten.

Manche Patient*innen nützen auch *Apps* wie „Neuronation“ und „Lumosity“^2^. Zu den Aufgaben von Ergotherapeut*innen zählt die klientenzentrierte Beratung, wie und wann das Training sinnvoll ist, wie viel Zeit man dafür aufwenden möchte/kann, wann das Nutzen der App angesichts der Belastung durch die Bildschirmbetrachtung sinnvoll ist (z. B. nicht vor dem Schlafengehen) und wie man die Ergebnisse des Trainings bewertet^3^ [47].

Klient*innen sollten darauf hingewiesen werden, dass eventuelle Schwankungen in der Leistung auch von Tagesverfassung, Erschöpfungszustand, vorangegangenen Tätigkeiten etc. beeinflusst werden können, da v. a. leistungsorientierte Klient*innen sich bei tageweise schlechteren Resultaten bei (auch App-basierten) Übungen oft enttäuscht zeigen [12, 35].

Jedes Training sollte grundsätzlich ein positiv besetztes Tool sein; viele Klient*innen berichten darüber, dass einzelne Aufgaben ansprechend sind und sie den spielerischen, Freude bringenden Charakter wie das Training insgesamt als positiv erleben. Dies ist bei sämtlichen Aufgaben der Fall, wenn die Klient*innen das Gefühl haben, etwas für sich selbst tun zu können, selbst zur Genesung beitragen zu können, und drückt die Bedeutung positiver handlungsbezogener Selbstwirksamkeitserfahrung und das große Anliegen von Klient*innen, zur Verbesserung der eigenen Situation beizutragen [16], aus.

Im Rahmen des kognitiven Trainings empfehlen Ergotherapeut*innen individuell für die jeweilige Person und deren Gesundheitssituation spezifische Übungen (vgl. [70]) bzw. verwenden Arbeitsblätter und Unterlagen aus diversen Therapiemanualen für die neuropsychologische Rehabilitation [47, 71].

Im Rahmen der Forschungsarbeit im PRECISE-Projekt hat sich gezeigt, dass erst in einer Phase der Genesung, in der Tätigkeiten im Alltag wieder leichter durchführbar sind, auch „traditionelles Hirnleistungstraining“ indiziert sein kann.

Betroffenen, die von „brain fog“ berichten, der ihrer Erinnerung, Konzentration und Planung subjektiv empfunden „im Weg steht“, können Ergotherapeut*innen Strategien vermitteln, die unter Berücksichtigung des Pacings Alltagskompetenzen schrittweise wiederaufbauen (vgl. [3]). Gerade wenn Patient*innen nach dieser ersten Phase der Genesung von Gedächtnisschwierigkeiten berichten, die den Alltag nicht unwesentlich beeinflussen, sei es im sozialen Kontext, im beruflichen Kontext oder auch bei der allgemeinen Alltagsorganisation, und diese als sehr belastend empfunden werden, ist es sinnvoll, mit ihnen Möglichkeiten interner und externer Gedächtnisstrategien zu besprechen, konkret auszuprobieren und anzuwenden [6, 11, 47]. Dies hilft nicht nur, die Bewältigung der Alltagsaufgaben zu erleichtern, sondern vermittelt den Patient*innen auch ein Erleben von Selbstwirksamkeit, da sie Tools erlernen, die sie selbstständig einsetzen und somit selbstbestimmter den Alltag gestalten können [47].

Analog zum Wiederaufbau motorischer Kompetenzen nach schwerem COVID-19-Verlauf müssen *Alltagstätigkeiten* (vom Zähneputzen bis zum Vorbereiten der Kinder auf den Schultag, vom Kaffeekochen bis zum Autofahren oder Benutzen von öffentlichen Verkehrsmitteln) in ihrer Reihenfolge wieder durchdacht und praktisch erprobt werden.

### Körperbezogene Aspekte

Ergotherapeutische Interventionen zur Behandlung von Symptomen in den Bereichen der Körperfunktionen und Körperstrukturen, die Biomechanik und Sensomotorik bedingen, sind eingebettet in interprofessionelle Behandlungsstrategien.

Bei Betroffenen von Folgen viraler Erkrankung mit SARS-CoV‑2 ist die Bedeutung des parasympathischen Nervensystems und dessen positive Beeinflussung (Vagotonus) zu beachten. Im Zusammenhang mit dieser Gesundheitssituation kann es zu einer Imbalance zwischen Sympathikus und Parasympathikus zugunsten des Sympathikus kommen: Klient*innen berichten beispielsweise von innerer Unruhe, reduzierter Schlafqualität, starkem Schwitzen, Übelkeit und Herzrasen sowie einer unregelmäßigen Herzfrequenz [72]. Ergotherapeut*innen beachten und adressieren diese Symptome in der Therapie auch dann, wenn sie diese nicht selbst beobachten, Betroffene diese aber im Hinblick auf ihren Alltag berichten. Zur Regulierung des Vegetativums können individuelle Übungen bzw. Verhaltensänderungen mit den Klient*innen für den jeweiligen Alltag besprochen und exemplarisch ausprobiert werden [12, 73]. Entspannungsübungen, Atemübungen, Meditation können auch zur unmittelbaren Selbsthilfe eingesetzt werden und positiv auf das Vegetativum wirken [47, 73].

Bei Lagewechsel kann es zudem zu starkem Schwindel, Herzrasen (hohe Herzfrequenz) und gleichzeitigem Blutdruckabfall kommen (posturales orthostatisches Tachykardiesyndrom [POTS]) [72]. Darauf muss bei alltagsorientiertem Training oder ADL-Training im Rahmen der Ergotherapie geachtet und ggf. u. a. mit kreislaufanregenden Maßnahmen vor Lagewechsel sowie einer längeren Warm-up- und Cool-down-Phase reagiert werden; zudem ist Rücksprache mit behandelnden Ärzt*innen unabhängig vom Therapiesetting wesentlich und jeweils aktuell verfügbare Forschungsergebnisse sind zu beachten (vgl. [72]).

In der Arbeit mit Betroffenen wurde deutlich, dass das Dehnen von Nerven auch bei Alltagstätigkeiten, wie z. B. Schuhe binden „Crashes“ (PEMs) provozieren kann. Das Vermitteln von neurodynamischen Übungen wurde insofern bis dato vermieden.

Gute Erfahrungen berichten Ergotherapeut*innen beim Einsatz *behutsamer Embodiment-Ansätze* und deren Transfer in Alltag und Bewegung.

In jedem Fall besteht hier dringender weiterer Forschungsbedarf, ehe Empfehlungen wissenschaftlich evidenzbasiert weitergegeben werden können. Gerade körperbezogene Interventionen, wie in diesem Abschnitt beschrieben, bedürfen unmittelbarer Kommunikation und Kooperation im interprofessionellen Versorgungs- und Forscher*innen-Team.

Noch im Prozess der Finalisierung dieses Dokuments werden laufend aktuelle medizinische und therapeutische Erkenntnisse gewonnen und publiziert, die insbesondere den Bereich posturales orthostatisches Tachykardiesyndrom (POTS) und „post-exertional malaise“ (PEM) betreffen. Im individualisierten therapeutischen Vorgehen gilt es, besonders feinfühlig und fachlich differenziert auf die Betroffenen in der evidenzbasierten Arbeit einzugehen. Dies betrifft insbesondere den Bereich des Energie- und Selbstmanagements wie auch den folgenden Abschnitt.

### Herzfrequenz und Sauerstoffsättigung (vgl. auch [1])

Im Zuge der sich prozessual entwickelnden Erkenntnisse zu den Auswirkungen einer SARS-COV-2-Infektion auf kardiopulmonale Parameter ist es auch hier wesentlich, sich am jeweils aktuellen medizinischen Wissensstand zu orientieren und die gesamte ergotherapeutische Intervention mit den behandelnden (Fach‑)Ärzt*innen individualisiert abzustimmen (s. auch [1]).

Im Rahmen der Ergotherapie im interprofessionellen Kontext ist Monitoring von Herzfrequenz und Sauerstoffsättigung auf die jeweilige *Alltagsgestaltung (Energiemanagement, Betätigungsprofil, Selbstmanagement)* zu beziehen (vgl. [74]). Das Durchführen einer Aktivität mit zu hoher Herzfrequenz und/oder zu niedriger Sauerstoffsättigung kann eine Symptomverschlechterung zur Folge haben. Daher kann es in der Ergotherapie je nach Möglichkeiten und Bedeutung dessen für den/die Klient*in sinnvoll sein, sie/ihn mit diesen Parametern und deren Beobachtung vertraut zu machen. Bei einer *verringerten Sauerstoffsättigung* kann es zu Atemnot oder einem Engegefühl im Brustraum kommen. Für die Klient*innen kann ein Abfallen der Sättigung jedoch auch unbemerkt verlaufen [5, 72]. Die jeweiligen Grenzwerte sind individuell mit den behandelnden Ärzt*innen abzusprechen.

Die Verwendung einer Pulsuhr oder eines Fingerpulsoxymeters im Alltag und/oder bei der Durchführung von Alltagsaktivitäten sollte interprofessionell abgesprochen im therapeutischen Setting vorbereitet werden. Konkrete Themen zur Selbstbeobachtung und Reflexion in der gemeinsamen Arbeit können u. a. Folgendes beinhalten:

- Bei welchen Tätigkeiten verändert sich meine Herzfrequenz und Sauerstoffsättigung?
- Welche Warnsignale kann ich während Alltagsaktivitäten erkennen?
- Wie viele Teilschritte einer Tätigkeit kann ich am Stück durchführen, ohne dass meine Sättigung zu stark fällt, meine Herzfrequenz steigt bzw. ich anschließend erschöpft bin, sich Symptome verschlechtern?
- Welche Möglichkeiten habe ich, die Tätigkeit so anzupassen, dass auch die relevanten Bioparameter (Werte) nicht entgleisen? (z. B. durch Durchführung im Sitzen, Pausen machen, Hilfsmittel verwenden) [47].

Für Betroffene ist es oft nicht einfach eine niedrigere Belastungsgrenze zu akzeptieren, da damit in der Regel deutliche Einschränkungen im Alltag einhergehen. Klient*innen leiden zumeist darunter, nicht mehr so zu funktionieren wie vor der Erkrankung, und die Anforderungen an sich selbst und den eigenen Körper stehen im Widerspruch zu den vorhandenen Ressourcen [72].

Der *Atem* als therapeutisches Mittel (in Kooperation mit Atemtherapeut*innen und Physiotherapeut*innen sowie körperorientierter Psychotherapie) bietet sich an, um diesbezügliche Aspekte zu adressieren.

Themen für die Ergotherapie können z. B. sein:

- Zu welchem Zeitpunkt am Tag können Atemübungen wohltuend sein?
- Welche Umgebung brauche ich dafür? (z. B. Atemübungen im Wald/an der frischen Luft)
- Wie kann ich gezielt *Atemübungen in Alltagsaktivitäten* einsetzen?

Eine häufig verwendete Technik ist die sog. „*Lippenbremse*“, mithilfe derer die Atemtiefe und -frequenz beeinflusst werden und eine Regulierung der Sauerstoffsättigung erfolgt [5]. Diese Art des Ausatmens lässt sich auch gut bei Alltagsaktivitäten, die für die Atmung herausfordernder sind, wie z. B. beim Bücken, beim etwas Aufheben, einsetzen. Außerdem führt das Anwenden der „Lippenbremse“ in Kombination mit atemerleichternden Körperstellungen, wie z. B. Kutschersitz, Torwartstellung, zur Atemerleichterung und Atemberuhigung (s. [74]).

In Kooperation mit den jeweiligen Fachärzt*innen, Psycholog*innen bzw. Psychotherapeut*innen ist im Hinblick auf das im Zusammenhang mit Folgen viraler Erkrankung mit SARS-CoV‑2 beschriebene *Hyperventilationssyndrom* (HVS) mit den Betroffenen zu erarbeiten, welche Warnsignale bei Alltagstätigkeiten beobachtet werden können (z. B. Kurzatmigkeit, Druck/Schmerz auf der Brust, Schwindel, Angst) und wie dieser Symptomatik Einhalt geboten werden kann [5].

Insgesamt ist es ratsam, als Ergotherapeut*in über Tools wie „Lippenbremse“, Atemtechniken, atemerleichternde Körperstellungen, sowie „*energy conservation techniques*“ (*Energiemanagement*) Bescheid zu wissen, um die Klient*innen diesbezüglich zu informieren, sie mit ihnen *in alltagsnahen bzw. Alltagssituationen* auszuprobieren und damit den Transfer in den Alltag zu erarbeiten [5].

In der Praxis berichten immer wieder Klient*innen auch von Symptomen wie Kurzatmigkeit, Engegefühl im Brustkorb, zu wenig Luft etc., ohne dass eine entsprechende pulmologische Diagnostik vorliegt. Auch hier ist es empfehlenswert, in Verbindung mit fachärztlicher Abklärung oben genannte Tools anzuwenden und einem pathologischen Atemmuster vorzubeugen [5, 47].

#### Ansatzpunkt UMWELT

Interventionen im Hinblick auf die sensorische Umwelt

Durch eine (vorübergehend) veränderte Reizschwelle beeinflussen sensorische Umweltreize das Wohlbefinden der Personen in den jeweiligen Situationen. Ergotherapeut*innen können Betroffene im Umgang mit sensorischen Reizen coachen/beraten, sodass Klient*innen für sich Umgebungen gestalten oder Strategien im Umgang mit starken sensorischen Reizen finden können (zum Beispiel: einkaufen – Wahl einer geeigneten Tageszeit, in der weniger Menschen einkaufen; Zug fahren – Sitzen entgegen der Fahrtrichtung, um die vorüberziehenden visuellen Eindrücke etwas zu verlangsamen, Verwenden von Hörstöpseln und Augenbinden, nach Möglichkeit Auswahl einer weniger frequentierten Reisezeit).

### Licht/Helligkeit und visuelle Reize

Einige Betroffene empfinden helles/direktes Licht (z. B. auch Sonne) als unangenehm bzw. belastend. Im Rahmen der Ergotherapie können Behandlungsräume entsprechend gestaltet werden (z. B. Vorhänge zuziehen, Lichtquellen anpassen).

Mögliche Empfehlungen für den Alltag der Patient*innen können dahingehend sein:

- Tragen eines Sonnenschutzes (z. B. Sonnenbrille, Hut, Kappe)
- in der Natur/an öffentlichen Orten: schattige Umgebungen bevorzugen, auswählen, suchen
- zu Hause: Beleuchtung mit indirektem Licht, kleinen Lichtquellen, warmem Licht; Vorhänge/Verdunkeln
- „minimalistische“ Gestaltung der Therapieräumlichkeiten/Wohnumgebung
- wenn Reduktion von Reizen nicht möglich ist als Strategie z. B. wenige, ausgewählte Objekte fokussieren.

### Lautstärke (Stimme, Umgebungsgeräusche)

Auditive Reize wurden von Betroffenen als mögliche Belastung/Stressor empfunden.

Besondere Herausforderungen ergeben sich, wenn die Patient*innen in einer geräuschvollen Umgebung wohnen. Auch in Gesprächssituationen ist das Filtern von auditiven Reizen eine oft beschriebene Schwierigkeit, für die es entsprechende Coping-Strategien braucht. Bei Vorliegen dieser Problematik empfiehlt sich ein Einzelsetting im Unterschied zum Gruppensetting.

In der Ergotherapie (unabhängig vom Setting) kann darauf geachtet werden, die eigene Stimme modulierend anzupassen, laute, unvorhersehbare Geräusche (wie z. B. das rasche Abreißen eines Blatt Papiers) zu vermeiden und Umgebungsgeräusche möglichst gering zu halten (z. B. Türen und Fenster schließen, in gemeinschaftlich genutzten Räumen „Bitte nicht stören“-Schild anbringen, Telefone stumm/ausschalten).

Weitere mögliche Empfehlungen für Patient*innen sind die Verwendung von Ohrstöpseln, das Nutzen von Kopfhörern (evtl. mit Noise-Cancelling, Over-Ear), auch ohne Musik zu hören, beispielsweise auch in öffentlichen Räumen wie auch bei der Benutzung von Verkehrsmitteln.

### Temperatur (zu heiß, zu kalt)

In Bezug auf das Temperaturempfinden scheint für Betroffene von Folgen viraler Erkrankung mit SARS-CoV‑2 moderate Wärme wohltuend zu wirken.

Mögliche Empfehlungen für Patient*innen:

- warme Socken, Hausschuhe
- Ankleiden mit Zwiebelprinzip – Möglichkeit, Kleidungsstücke, je nach Temperatur an- oder auszuziehen
- Verwenden von Wärmekissen, Heizdecken
- Fußbäder (im Sommer kaltes Fußbad zum Abkühlen, im Winter warmes/heißes Fußbad zum Aufwärmen)
- Regulieren der Zimmertemperatur

Weitere physikalische Maßnahmen, wie z. B. Kryotherapie, werden in Kur- und Rehazentren bereits zusätzlich angeboten.

### Olfaktorisch

Sowohl die Sensibilität (Empfindsamkeit) als auch die Rezeptivität (Empfänglichkeit) für Gerüche sind in der therapeutischen Situation wie auch im Alltag der Betroffenen zu berücksichtigen. Wissenschaftliche Studien zur Wirkung olfaktorischer Reize auf das parasympathische bzw. limbische System könnten dazu weitere Ansatzpunkte geben.

### Zeit als Umweltfaktor

Der Umgang mit verfügbarer chronologischer Zeit stellt sich für Betroffene von Folgen viraler Erkrankung mit SARS-CoV‑2 anders als gesellschaftlich verankert dar. Die Notwendigkeit, mit weniger Energie als vor der Infektion den jeweiligen Alltag zu gestalten, wirkt sich direkt, für die Betroffenen und ihr Umfeld unmittelbar und oft herausfordernd, aus. Die Veränderung und notwendige Neu-Entwicklung von Handlungsroutinen, das Orientieren am aktuell Möglichen, das Nutzen der dafür individuell besten Tageszeit (je nach Tagesverfassung) sind nur einige wenige Aspekte, die hier in einer postakuten Rehabilitationsphase ergotherapeutisch zu adressieren sind. Auf andere Weise stellen sich die Themen im Kontext beruflicher Re-Integration dar: Geschwindigkeit, die in unseren Arbeitswelten erwartet wird, beruht u. a. auf verlässlicher motorischer Planung und Handlungsplanung, die auch bei verringerter Symptomatik ergotherapeutisch im Hinblick auf die konkrete Arbeitssituation, -umwelt, -aufgabe, -kapazität mithilfe von u. a. Handlungsperformanzanalysen, Umweltanalysen und Beratung von Arbeitgeber- wie -nehmer*innen zu analysieren und zu erweitern ist. Hilfreiche Frage kann hier, auch anhand eines Betätigungsprofils, sein, was wann wo mit wem zu tun möglich und sinnvoll zu priorisieren ist. Auch die Balance unterschiedlicher Anforderungen in Tätigkeiten im zeitlichen Tages- und Wochenverlauf sind ergotherapeutisch relevante Themen in der Arbeit mit den Betroffenen und deren Angehörigen.

### Leben in und mit der sozialen Umwelt

Die soziale Umwelt spielt für die Bedeutung der und den Umgang mit der aktuellen Gesundheitssituation eine wesentliche Rolle. Ergotherapeut*innen bedenken, involvieren, beraten, coachen und stärken Personen im Alltag der Betroffenen in ihrer Handlungsrolle als Angehörige/Bezugspersonen direkt und indirekt. Dies dient der Bewältigung des Alltags mit Implikationen und Veränderungen durch die nach der COVID-Infektion vorhandenen Herausforderungen.

Gerade das Anerkennen gegenwärtiger Grenzen und das stufenweise (Wieder‑)Aufbauen von Möglichkeiten und Grenzen seitens der Betroffenen UND ihres sozialen Umfelds ermöglichen Pacing, Graduieren von Tätigkeiten, damit auch Vermeidung von „Crashes“/PEMs und Stärkung von Resilienz und (wie genannt, stufenweisen) Wiederaufbau von alltagsrelevanten Kompetenzen. Bei Bedarf sind durch Ergotherapeut*innen und das interprofessionelle Team Vernetzungsgespräche bzw. Coaching-Angebote auch für das soziale Umfeld anzubieten.

Betroffene haben oft das Bedürfnis nach Ruhe und alleine sein; direkte Begegnungen, Telefonate, Kontakte werden vorübergehend nicht (mehr) als wohltuend und stärkend, sondern als anstrengend erlebt (s. auch sensorische Umwelt, sensorische Regulation). Dies betrifft den unmittelbaren Kontakt mit anderen Menschen mehr als die innere Verbundenheit mit diesen selbst. In der konkreten ergotherapeutischen Begleitung berichteten besonders auch bis vor der Erkrankung sehr kinderliebende Betroffene bedrückt, insbesondere jüngere und bewegungsfreudige, aktive Kinder wegen der intensiven sensorischen Stimuli (in dieser Phase) nicht mehr auszuhalten [12].

Für Betroffene kann es herausfordernd sein, gegenüber Freund*innen, Studien- oder Arbeitskolleg*innen, insbesondere auch ihre*n Partner*in, eigenen Kindern, Eltern bzw. Großeltern die eigene geringere Belastbarkeit zu thematisieren und auch trotz der Einschränkungen für beide Seiten positive Beziehungserlebnisse und ein adäquates Bindungsempfinden zu ermöglichen. Diesen Prozess gilt es ergotherapeutisch zu begleiten [12, 64]; hilfreich könnten dabei u. a. zum Thema passende Kinderbücher sein [47].

Zwei Tätigkeiten (wie z. B. reden und essen, essen und zuhören) gleichzeitig auszuführen ist für viele oft zu anstrengend; dies hat unmittelbare Auswirkungen auf die soziale Partizipation (z. B. Einnehmen von Mahlzeiten alleine im familiären wie, später, im Arbeitsumfeld).

Im Rahmen ergotherapeutischer Beratung ist es insofern auch wesentlich, konstruktive Kommunikationsmöglichkeiten der Patient*innen mit ihrem jeweiligen sozialen Umfeld (Familie, Freunde, Bekannte, Arbeitskolleg*innen, Arbeitgeber*innen) im Hinblick auf ihre Kapazitäten und aktuellen Grenzen zu adressieren.

Viele Patient*innen haben das Gefühl, sich für ihre (noch relativ unbekannte) Gesundheitssituation rechtfertigen oder erklären zu müssen, bzw. erleben „rein psychische“ Attribuierungen dieser; viele haben eine Aufklärungsrolle bezüglich des Krankheitsbildes Folgen von viraler Erkrankung mit SARS-CoV‑2 (bzw. Post/Long-COVID) inne. Auch die Problematik mit *Wortfindungsstörungen*, die viele berichten, wirkt sich in der Kommunikation sehr belastend aus. Diesbezüglich ist das mit den Betroffenen *gemeinsame Erarbeiten von Verhaltensstrategien und Kommunikationsmöglichkeiten* wichtig [3–5, 12, 47].

### Coaching/Beratung zu Umweltgestaltung und Hilfsmitteln

Umweltanalysen, um die Auswirkungen auf „Occupational Engagement“ in alltäglichen Handlungen durch Strategien und Adaptierungen zu fördern, gehört zu den Kernkompetenzen und -aufgaben von Ergotherapeut*innen [3, 48]. Je nach Setting inkludiert dies auch die Versorgung mit jeweils indizierten Hilfsmitteln, das Eingehen auf die konkreten Lebensumwelten der betreffenden Person einschließlich ihrer sozialen Umwelt in Familie, Freundeskreis, Arbeitskontext, Community [55].

### Arbeit mit Angehörigen (vgl. auch [3])

Das unmittelbare familiäre Umfeld beeinflusst die Betroffenen von Folgen viraler Erkrankung mit SARS-CoV‑2 im Hinblick auf Alltagsgestaltung, Handlungsrollen, Betätigungsbalance, Handlungsroutinen und -muster und ist gleichzeitig selbst von der Gesamtsituation betroffen. Der gemeinsame Alltag und erprobte Aufgabenaufteilungen, liebgewonnene (gemeinsame) Tätigkeiten und Gewohnheiten sind durch die deutlich geringere Belastbarkeit neu zu entwickeln und zu meistern. Im Rahmen der Ergotherapie ist es wichtig, Angehörige in ihren Handlungskompetenzen als (Mit‑)Pflegende zu stärken, auf ihre Betätigungsgesundheit einzugehen und sie dahingehend zu beraten, auf gemeinsam als wertvolle, wohltuende Momente, Erlebnisse und geteilte Lebenszeit auch gegenwärtig in der Alltagsgestaltung zu achten. Angehörigeninformation, -beratung und -coaching sind mit dem Einverständnis der Patient*innen selbst wesentliche Bausteine in der interprofessionellen Versorgung und handlungs- und alltagsorientiert Kernkompetenz von Ergotherapeut*innen [7, 48].

### Bedeutung der institutionellen Umwelt

Betroffene von Folgen viraler Erkrankung mit SARS-CoV‑2 stoßen – auch angesichts der noch wenig bekannten Implikationen auf Handlungsfähigkeiten – oft auf Unverständnis für ihre Situation. Gleichzeitig sind sie mit existenziell bedrohlichen Situationen angesichts ihres Krankenstandes, ihrer (drohenden) Kündigung oder ihrer aktuellen Unfähigkeit, ihre Aufgaben in Familie, Ausbildung, Gesellschaft wahrzunehmen, konfrontiert.

Mit ihrer Symptomatik bedeuten notwendige Behördenwege, die Erledigung von Formalitäten, noch nicht gelöste Situationen auf sozialversicherungsrelevanter bzw. rechtlicher Ebene oftmals Ansatzpunkte für die Ergotherapie. Dabei kann es – in Kooperation mit anderen Berufsgruppen wie Soziale Arbeit, Gesundheits- und Krankenpflege, Ärzt*innen, Hilfsdiensten – um Themen von der Essensversorgung bis hin zur Wiedereingliederung in den Arbeitsmarkt gehen.

#### Ansatzpunkt HANDLUNG

In der Ergotherapie stehen Handlungspotenziale, Handlungsinteressen, Handlungsmöglichkeiten, Handlungskompetenzen von Menschen im Vordergrund. Bei Betroffenen von Folgen viraler Erkrankung mit SARS-CoV‑2 geht es u. a. darum, Handlungsroutinen, die gesundheitsförderlich sind, zu entfalten, aktuelle Handlungsinteressen zu identifizieren und „jeden Tag etwas Freudvolles zu erleben/tun“ [11].

International hat sich die „3- bzw. 4‑P-Regel“ für das Pacing als Handlungs- und Verhaltensempfehlung etabliert: priorisieren, planen, Pausen einplanen, positiv bleiben (vgl. [50, 51]).

Pacing bedeutet im Alltag nicht alleine das Graduieren und die Dauer der Tätigkeiten selbst, sondern auch das Bedenken des Gewichts von Gegenständen oder auch der Anforderungen von Wegstrecken, die zurückzulegen sind.

Wesentliches ergotherapeutisches Werkzeug stellt dafür die sog. Handlungsperformanzanalyse dar – vor, während und nach dem konkreten Tun der Patient*innen bei der Durchführung von für sie bedeutungsvollen (wichtigen, notwendigen, sinnstiftenden) Tätigkeiten.

Ergotherapeut*innen geben professionelle Unterstützung bei Handlungen; deren Spektrum betrifft Aktivitäten im Bereich der Selbstversorgung wie duschen, kochen, Haushalt führen, erledigen bürokratischer Aufgaben (Ausfüllen von diversen Anträgen im Zusammenhang mit der Erkrankung wie Behindertenpass, Beantragung von Reha-Geld, …), Therapie, Beratung und Coaching hinsichtlich beruflichem Wiedereinstieg sowie die Gestaltung von freier und erholsamer Zeit. Die Klient*innen werden zudem darin ergotherapeutisch begleitet, neue Handlungsrollen anzunehmen, für sich zu finden und darin aktiv zu werden.

Um die Handlungsmöglichkeiten zu erweitern, beraten Ergotherapeut*innen im Hinblick auf individuell sinnvolle Hilfsmittel, die nach Möglichkeit miteinander organisiert werden können, ebenso wie hinsichtlich ökonomischer Körperpositionen im Tun (auch im Sinne des Energiemanagements).

Ein besonderes, von neuseeländischen und australischen Berufskolleg*innen bedientes Feld sind Abklärungen, Beratung und Interventionen im Zusammenhang mit Autofahren. Für Betroffene von Folgen viraler Erkrankung mit SARS-CoV‑2 ist es entsprechend Praxisevidenz potentiell herausfordernd, alle relevanten Aspekte visuell zu erfassen, die verschiedenen Modalitäten mit passender motorischer Antwort zu koordinieren und damit rechtzeitig zu reagieren. In dem Zusammenhang müsste über (interprofessionelles) Fahrsicherheitstraining sowie ergotherapeutisch gecoachte Fahrstunden nachgedacht werden.

### Dokumentation und Evaluation

Wie oben bereits im Hinblick auf prozessuale Evaluation (auch gemeinsam mit den Klient*innen [23] und im interprofessionellen Team) und ggf. Adaptierung gesetzter Ziele beschrieben, wird zusätzlich dazu in der niederländischen „Guideline Occupational Therapy for clients with POST-COVID Syndrome“ [5] vorgeschlagen, nach 3, 6 und 12 Monaten periodische Evaluation routinemäßig durchzuführen. In jedem Fall gilt es, auf aktuelle Anliegen der Klient*innen responsiv einzugehen, insbesondere durch die Schwankungen in deren biopsychosozialer Gesundheitssituation.

## Berufliche (Re‑)Integration

Die Vorbereitung und Begleitung des Wiedereinstiegs ins bezahlte wie unbezahlte Arbeitsleben gehören zu den Kernaufgaben von Ergotherapeut*innen für unterschiedliche Zielgruppen und haben sich im Rahmen des PRECISE-Projekts analog zu internationalen Publikationen auch für Betroffene von Folgen viraler Erkrankung mit SARS-CoV‑2 bewährt [3, 20, 54, 68, 75–78].

Wesentlich ist auch hier wieder das stufenweise Vorgehen. Brehon et al. [78] betonen, dass insbesondere flexible, graduelle „Return to work“-Pläne Menschen mit Folgen viraler Erkrankung mit SARS-CoV‑2 in ihrer Rückkehr zum Arbeitsplatz unterstützen.

*Im teletherapeutischen Setting* [79, 80], das auch hier relevant sein kann, stehen ergotherapeutisches Coaching und Beratung/Begleitung im Vordergrund; gerade bei Betroffenen, die geografisch weitere Strecken oder aber im Hinblick auf die Reizumgebung fordernde sensorische Umwelten für einen Weg zur Therapie überwinden müssten, ist dies bis hin zur Phase beruflicher (Re‑)Integration sehr zu empfehlen. Auf diese Weise kann der Fokus der Interventionen auf das konkret Dringliche gelegt und die verfügbare Energie genau für diese Inhalte genutzt werden. Dies ist insofern bedeutsam, als auch kognitive und emotionale Prozesse Energie kosten.

Entscheidend für eine gelingende berufliche Reintegration sind u. a. auch Verständnis und Akzeptanz der Problematik im Arbeitskontext durch Arbeitgeber*in und das jeweilige Team. Gefahren, die auch Rückschläge bringen können, bestehen u. a. in Überforderung, Mobbing und (drohendem bzw. befürchtetem) Arbeitsplatzverlust.

Ergotherapeut*innen bringen sich hier beratend, coachend, informierend, Umwelt gestaltend und koordinierend ein.

Sie unterstützen in der Kommunikation mit Arbeitgeber*in und Kolleg*innen und koordinieren professionelle Unterstützungsangebote klientenzentriert und kontextsensibel.

Arbeitstherapie als ein historischer Ursprung der Ergotherapie bereitet auf den Transfer in den beruflichen Alltag vor. Dem voraus geht in der Regel eine vorbereitende Phase im häuslichen Setting. Dabei gilt als Orientierung, dass zuerst der Alltag im häuslichen Umfeld (z. B. Aktivitäten des täglichen Lebens) gelingt, ehe die berufliche Tätigkeit wieder ausgeübt werden kann.

Wenn von Betroffenen gewünscht und seitens der Kostenträger unterstützt, kann ergotherapeutische Begleitung durch den Prozess des Krankenstands bis zur Wiederaufnahme der Arbeit (mit schlussendlich einem Arbeitspensum wie vor der Erkrankung) angeboten werden.

Ergotherapeutische Expertise ist im Sinne eines Case Managements nützlich: handlungsorientierte Einschätzung der arbeitsbezogenen Leistungsfähigkeit mit Betroffenen, Vernetzung mit Arbeitgeber*innen, Unterstützung der Patient*innen bei Gesprächen/Zielabklärung/Planung der Rückkehr an den Arbeitsplatz, Vernetzung mit/Information zu unterstützenden Dienstleistungen wie BBRZ, fit2work, arbas u. a.

Ergotherapeut*innen begleiten den Prozess der Rückkehr an den Arbeitsplatz bzw. den der beruflichen Neuorientierung mit fachspezifisch handlungs-, ressourcen- und klientenzentriertem, kontextsensiblem Zugang [8]; Arbeit im Netzwerk und gegenseitiges Abstimmen sind dabei auch insofern essenziell, als in dem Zusammenhang mehrere Personen (beruflich wie privat) und Behörden beteiligt sind. Es gilt, Patient*innen Sicherheit und niederschwellige Hilfe zu geben; sie verstehen nicht immer alle (verbalen) Informationen, sind kognitiv ermüdbar [68], und haben wenig Kraft, Gespräche zu initiieren bzw. aktiv zu führen. Auch dahingehend ist Ressourcenorientierung beizubehalten und zu fördern, Zuversicht zu stärken.

Am Arbeitsplatz selbst und für Arbeitgeber*innen wie Arbeitnehmer*innen führen Ergotherapeut*innen Aktivitäts- und Anforderungsanalysen, Umwelt- inklusive Materialanalysen durch. Sie erstellen berufsbezogene Betätigungsprofile in Verbindung mit Pacing und Energiemanagement-Tools für die Patient*innen und beraten hinsichtlich Ergonomie, Hilfsmittelversorgung und Energiesparpotenzialen u. a. durch passende Arbeitsorganisation und Pausenmanagement.

## Ergotherapeutische Arbeit im Netzwerk, interdisziplinäre und interprofessionelle Zusammenarbeit

In der ergotherapeutischen Arbeit im Rahmen des PRECISE-Projekts zeigte es sich als wesentlich, den Betroffenen und bei Bedarf auch ihren Angehörigen Orientierung in der sog. sozialen Netzwerklandkarte zu geben.

Zugang zur Gesundheitsversorgung und zu Hilfsdiensten (wie Ergotherapie, Essen auf Rädern, konkrete Anlaufstellen für Betroffene) müssen zielgruppengerecht verfügbar sein bzw. werden, mit Folgen viraler Erkrankung mit SARS-CoV‑2 (gesundheitsbezogen) fachlich vertrautes Case Management ist dabei eine noch weiter auszubauende Notwendigkeit [81].

Das Wissen um lokale, unterstützende Gruppen oder Online-Foren [20], Wissen um Anlaufstellen z. B. bei finanziellen Schwierigkeiten [20] ist im Sinne dieses Case Managements für die jeweiligen Ergotherapeut*innen notwendig; dieses gilt es bedarfs- und bedürfnisorientiert auch an die Betroffenen weiterzugeben und damit ihre Gesundheitskompetenzen (Health Literacy) weiter zu stärken.

Wie bedeutsam interprofessionelle und interdisziplinäre Kooperation und Kommunikation sind [53–55], beschreiben u. a. auch Wolf et al. [19] und warnen vor entstehenden Unsicherheiten, wenn mehrere Anlaufstellen zeitgleich aufgesucht werden und es eine Fülle an (möglicherweise nicht übereinstimmenden) Empfehlungen gibt.

Sämtliche ergotherapeutische Kernkompetenzen sind in der Arbeit im interprofessionellen, interdisziplinären Netzwerk für Menschen Folgen viraler Erkrankung an SARS-COV‑2 notwendig [48, 81, 82].

## Kinder und Jugendliche

Grundsätzlich sind die in den oberen Abschnitten beschriebenen Ansatzpunkte auch für Kinder und Jugendliche relevant.

Bis dato liegen für diese Altersgruppe noch kaum wissenschaftliche Evidenzen spezifisch für die Folgen viraler Erkrankung mit SARS-CoV‑2 vor, was ergotherapeutische Interventionen betrifft. Das Vorgehen entsprechend ergotherapeutischen Best-practice-Kriterien [8, 9, 11, 83] und der kritische Transfer von für Erwachsene hilfreichen handlungsorientierten Interventionen ist daher aktuelle Ausgangslage (vgl. auch [3, 84]). Auch bei Kindern und Jugendlichen steht die Klient*innenzentrierung im Vordergrund, im Sinne eines personalisierten und partizipativen Ansatzes, bei dem Entscheidungen gemeinsam mit den Betroffenen getroffen werden [3, 85]. Ebenso bedeutsam sind gute Kommunikation und Zusammenarbeit im multiprofessionellen, interdisziplinären Team und mit den Eltern. Wolf [85] beschreibt gelingende Kommunikation zwischen Klient*innen, Therapeut*innen und Ärzt*innen als Schlüsselfaktor.

### Lebensumwelt Schule und Ausbildung/Arbeit

Kinder und Jugendliche haben aufgrund der Symptomatik der Folgen viraler Erkrankung mit SARS-CoV‑2 Schwierigkeiten, in die Schule bzw. in die Lehrstelle zurückzukehren. Diese Problematik hat auch mittel- und langfristig Konsequenzen für Kinder und Jugendliche [1, 85]. Im Zuge der *Stärkung des Selbstmanagements* können u. a. folgende Informationsseiten herangezogen werden:

https://de.longcovidkids.org/; https://longcovidkids.ch/; https://www.yourcovidrecovery.nhs.uk/children-and-young-people-with-covid/

Der National Health Service (NHS) [86] bietet Empfehlungen für Kinder und Jugendliche sowie für deren Eltern und Angehörigen zu Themen wie *Aktivitäten des täglichen Lebens, Ernährung, Schlaf* und *Gefühle*. Als international empfohlene Leistungen für betroffene Kinder und Jugendliche mit Folgen viraler Erkrankung mit SARS-CoV‑2 fasst Wolf [85] (*im Folgenden adaptiert*) zusammen:

- Reintegration in einen gesundheitsförderlichen Alltag, ins soziale Leben
  - Tipps für Selbst- und Co-Management
  - Anpassungen/Graduierungen von sportlichen Aktivitäten, Auswahl möglicher sozialer Aktivitäten mit Familie, Freund*innen
  - Abstimmung mit Gesundheitsprofessionen, Sozialer Arbeit, Pädagog*innen, Eltern und Patient*innen
- Unterstützung für Eltern/Bezugspersonen und Geschwister
- Beratung, Coaching und Begleitung im Prozess der Reintegration in Schule, Ausbildung oder Arbeit: entsprechende Anpassungen, z. B. graduelle Rückkehr, Erholungszeiten, Koordination/Kommunikation mit Pädagog*innen/Schule, Gesundheitsprofessionen, Sozialer Arbeit, Eltern
- Kooperation mit Sozialer Arbeit im Hinblick auf finanzielle Unterstützungen (z. B. Pflegeurlaub, Unterstützungsmöglichkeiten seitens Arbeitgeber für Eltern)
- Netzwerkarbeit im Gesundheitswesen: u. a. Kooperation mit Allgemeinmediziner*innen sowie Kinderfachärzt*innen; Kooperation in und mit Akut- oder Spezialkliniken sowie multidisziplinären Versorgungszentren; bedarfsorientiert mit Psychotherapie, Physiotherapie, Diätologie u. a. m.

Dieses Dokument basiert, wie eingangs erwähnt, auf – bis zum Datum dessen Freigabe durch Ergotherapie Austria – verfügbarer Evidenz und soll evidenzbasierte Praxis im Sinne Sackett’s zum Wohle betroffener Klient*innen unterstützen. Die konkrete Auswahl und Passung ergotherapeutischer Interventionen liegt in der Verantwortung der/des behandelnden Berufsangehörigen und muss sämtliche Aspekte professionellen Reasonings in Verbindung mit ständig wachsendem, jeweils aktuell verfügbarem Wissen zur bestmöglichen Gesundheitsversorgung heranziehen.

## Footnotes

1 Persönliche Kommunikation mit Elisabeth Semotan-Rigler, Arbeitskreis „Ergotherapie post Covid“ von Ergotherapie Austria. 2022 Okt 5.

2 Persönliche Kommunikation mit Elisabeth Semotan-Rigler, Arbeitskreis „Ergotherapie post Covid“ von Ergotherapie Austria. 2022 Okt 5.

3 Persönliche Kommunikation mit Elisabeth Semotan-Rigler, Arbeitskreis „Ergotherapie post Covid“ von Ergotherapie Austria. 2022 Okt 5.
